# The Importance of Heating Unit Operations in the Food Industry to Obtain Safe and High-Quality Products

**DOI:** 10.3389/fnut.2022.853638

**Published:** 2022-04-26

**Authors:** Carmen C. Tadini, Jorge A. W. Gut

**Affiliations:** ^1^Department of Chemical Engineering, Escola Politécnica, Universidade de São Paulo, São Paulo, Brazil; ^2^Food Research Center FoRC, Universidade de São Paulo, São Paulo, Brazil

**Keywords:** processing, heat treatment, preservation, shelf-life, pasteurization, food safety

## Abstract

Civilization has begun around 3,500 BCE in Mesopotamia and the realization by people that they could manipulate food to preserve it, through sun drying, fermentation, freezing in the snow, or cooking by fire, was an important factor for the nomadic humans to start settling. Food by nature is subject to spoilage and the application of any kind of preservation method enables storage and weighted consumption. Throughout human history, many techniques have been developed and improved such as heat treatment, drying, freezing, extraction, mixing and the use of preservatives, among others. In the food industry of the modern world, each technique is implemented through sequential steps, known as unit operations. This opinion paper presents an overview of the main heating unit operations used in the food industry, highlighting their benefits to converting raw materials into palatable products with high quality and safe for consumption. Examples are presented to illustrate how several food products available in the market were submitted only to physical transformations based on scientific knowledge. However, there is a range of intensity in physical processing and the applied energy level depends on the nature of the food, target microorganism, storage conditions, type of packaging, and desired shelf-life. The importance of food safety is stressed since processed foods have been criticized for confusion between nutritious values and processing steps. There are still many challenges to the food industry to design the process in optimal conditions for food quality and with less environmental impacts and novel thermal and non-thermal technologies have been studied and implemented.

## Introduction

Before the beginning of civilization, our ancestors were nomads that gathered and hunted for food. Gastrointestinal-related diseases were probably common because of the consumption of contaminated, spoiled, or poisonous food. We can say with some reserve that with the advent of fire, cooking largely reduced microbial and toxin risks in food consumption. However, because of the limited conditions of cooking, these risks could not be completely avoided. Moreover, we can assume that our ancestors perceived that cooking made food more palatable, mainly meat and fish (1).

As time progressed, historical evidence are more likely to be found nowadays about foodborne diseases and how the knowledge of preservation has improved. For instance, examination of Egyptian mummies showed evidence of food and water-borne parasites. Curiously, for the preservation of bodies through mummification, Egyptians employed salts. Salting is still today a method used for food preservation, as in ham and some kinds of sausages. Nowadays, it is known that the physical principle is to reduce water activity, as in freezing or drying (1).

During medieval times, to tamper with the poor quality of the food, people used spices like nutmeg to adulterate the smell and taste of the spoiled meat. This spice became so popular, that it is used in the present days in hot dogs and sausages.

Only from the 16th Century, the origin and scientific basis of food-borne diseases gradually began to be understood, and consequently how to better preserve foods. For instance, A. van Leeuwenhoek (1632 – 1723), a self-taught Dutchman, constructed the microscope with which he could observe bacteria. Leeuwenhoek has been called the “father of microscopy” (2).

During the industrial revolution (18th Century), providing food for urban workers resulted in a large realignment of production and trade, in particular, agricultural production, food processing, and trade concentrated in enterprises (1). As a consequence, a great deal of food poisoning occurred simply because there was no knowledge about the mechanisms of food preservation.

We invite the reader to imagine, based on these historical events, how food diseases and the knowledge and development of food processing had an impact on human history.

### Pasteur’s Legacy

It is not an exaggeration to state that Pasteur’s findings were the beginning of what we know today as heat treatment, an important Unit Operation for the preservation of food. Before him, in the middle of the 18th Century, it was accepted that the application of heat could delay the spoilage of some foods. However, this was an empirical finding, since the concept of microbial spoilage was not yet known. Nicholas Appert (1749–1841), a Parisian confectioner, developed a method to preserve some types of food, which is now known as appertization, and we can state that it was the first process of canning, although Appert used glass bottles instead of cans.

About 100 years after Appert’s findings, Pasteur showed that temperatures around 60°C for a short time effectively eliminated spoilage microorganisms in wine. He demonstrated that microorganisms cause diseases and, beyond his contribution to the preservation of wine and beer, he discovered how to make vaccines from weakened or attenuated microbes. The heat treatment developed by Pasteur, known as pasteurization, was adopted by milk producers, at the end of the 19th Century, to eradicate most of the foodborne illnesses.

Due to Pasteur findings, almost globally, governments have passed laws requiring the pasteurization of milk used by the dairy industry, for any kind of consumption. A typical temperature/time combination for pasteurization is 72°C for 15–20 s, to eliminate pathogens. It is a magnificent example of how important are the fundamentals of food science and adequate use of Unit Operations (thermal processing), to provide safe food for consumers.

In summary, this opinion paper has the objective to highlight the importance of food processing at different levels for human health. Specifically, we highlight the physical method of thermal processing, quite commonly applied by food processors to provide safe food to the population. Moreover, we show how important it is to understand the Unit Operations that are employed in food processing at controlled conditions to offer food products that are safe and have high quality.

## Unit Operations

There are many reasons to process food, in addition to increasing its shelf life, as to make it more palatable and digestible, to inactivate naturally existing toxins and enzymes, or even to produce new products, such as ice cream. These goals are achieved through specialized processes.

Given its diversity, the study of processes involving food can be considered a challenge. But a careful analysis shows that these processes, although distinct and complicated, can be broken down into a small group of basic operations. Many of the traditional unit operations used in the industry have been part of culinary practices for ages, such as heating, freezing, washing, cutting, mixing, baking, curing, cooking, among others. The main difference is that in the industry the quality of the raw materials, the processing conditions, as well as packaging and storing, are regulated to produce safe and healthy products. Recently, Knorr and Augustin (3) properly compared the principles underlying the processes used in the industry and culinary practices and we recommend reading their review.

As already mentioned, in the food industry many transformations involve heating or cooling, such as bread baking, meat freezing, or oil refining. In such cases, you must first consider how much heating or cooling is required and then how the heat exchange should be conducted. The fundamentals involved in these physical processes are the same, whether increasing (heating) or decreasing (cooling) the temperature. Those are examples of unit operations that involve only physical transformation, in this case, heat transfer. In general terms, a unit operation can be defined as a basic operation in a process. Conversely, a process can be understood as a set of unit operations that, starting from a certain raw material, results in a specific product, that is safe for consumption, stable under storage, and palatable (4, 5).

Unit Operations are basically organized into operations of material transport, heat transfer, mass transfer, or a combination of them. The principles behind many Unit Operations used in industry are similar to those practiced in households or restaurants. For example, operations involving size reduction or mixing are common practices both in the food industry and culinary preparation. Others involve heat transfer, such as heating or chilling foods, or mass transfer, as filtration or extraction. Drying is a unit operation involving heat and mass transfer simultaneously.

In the next session, we highlight a Unit Operation based on heat transfer, the thermal processing, because of its great importance from the point of view of public health.

### Heating Unit Operations

For food processors, thermal processing is an important heat transfer unit operation and it can be conducted under batch or continuous conditions. Thermal processing is mainly divided into pasteurization (mild heat treatment with temperatures below 100°C) and commercial sterilization (temperatures above 100°C). Thermal processing involves heating, holding, and cooling steps. In pasteurization, there is a partial destruction of microbes, although ensuring food safety, while in sterilization, the inactivation of microbes is more intense, including spores.

It is important to say that canned foods are included in this kind of thermal processing and, depending on the pH, the heat conditions are differently applied. In general, for low-acid foods, like milk, the main objective of pasteurization is the inactivation of microorganisms of public health importance.

The pasteurization as a unit operation was redefined by the USDA as “any process, treatment, or a combination thereof, that is applied to food to reduce the most resistant microorganisms of public health risk under normal conditions of distribution and storage” (6).

Let us better understand the differences between HTST and UHT, concerning the heat treatment of milk. Fortunately, all common pathogenic organisms likely to occur in milk can be killed by a relatively mild heat treatment that has only a very small effect on the physical and chemical properties of milk. The most resistant pathogen is the tubercle bacillus (T.B.), which can be inactivated by heating milk to 63°C and holding for 30 min. Today in the dairy industry, the high-temperature short-time, known as the HTST process, is widely applied at 72°C for at least 15 s (time equivalent to 63°C for 30 min) to destroy all pathogens in milk. Since pasteurization is a mild heat treatment, after the process the product needs to be stored under refrigeration.

**FIGURE 1:**
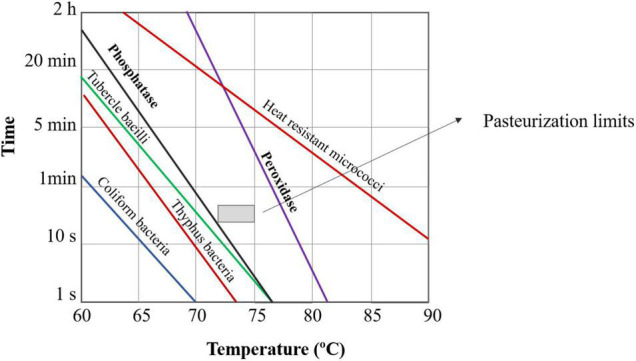
Effect of temperature and holding time on the lethality of common microorganisms and enzymes current in raw milk, indicating the pasteurization conditions [adapted from Bylund et al. (2)].

Since the Kay and Graham announcement in the middle 1930s, the activity detection of the phosphatase enzyme in pasteurized milk indicates a non-adequate thermal process. This enzyme is always present in raw milk and it is considered a process target. In Figure 1, it is shown the effect of different temperature-holding times on the common microorganisms and enzymes present in raw milk. If the process is conducted within the pasteurization limits indicated in Figure 1, the phosphatase test is negative, indicating an adequate process. However, the peroxidase test should be positive, which ensures that there was no over-processing of milk, preserving its nutritious value.

Because of the presence of pathogens, it is not safe to consume raw milk and, in most countries, it is forbidden to commercialize raw milk for consumption.

The combination of the temperature and holding time is very important to determine the intensity of the heat treatment. Intense heat treatment of milk is desirable from a microbiological point of view, however other undesirable effects can occur, such as a change in taste and nutritional value. Therefore, to offer a safe and nutritious product, food scientists and processors have researched for the more efficient technologies or more rigorous control methods, resulting in a safe product with high-quality retention.

It is well established that different combinations of temperature and holding time can lead to similar effects on microbial or enzymatic inactivation. And it is known that if a higher temperature is applied, a shorter time is needed for the same microbial inactivation (see Figure 1). For comparison, in Table 1 the main categories of the heat treatment applied to milk are shown. HTST is an abbreviation for High-Temperature Short-Time, conditions widely applied to pasteurize milk in continuous processing. In this treatment, as already mentioned, the phosphatase activity test is used to verify the proper pasteurization.

**TABLE 1:** The different combinations of temperature and time of heat treatments applied by the dairy industry to milk (2).

Heat treatment	Temperature (°C)	Time
Thermisation	63–65	15 s
LTLT pasteurization	63	30 min
HTST pasteurization	72–75	15–20 s
Indirect UHT	125–138	2–4 s
Direct UHT	135–140	a few seconds
Sterilization in container	115–120	20–30 min

UHT is an abbreviation for Ultra High Temperature, that can be applied at the dairy industry by two alternative techniques: indirect heating in heat exchangers or by steam injection followed by cooling by expansion under vacuum. The last is widely applied today. Both heat treatments (HTST and UHT) are conducted by temperature increase, but with different intensities. It is important to emphasize that these Unit Operations are conducted only applying heat and no preservatives are used to preserve the milk.

**FIGURE 2:**
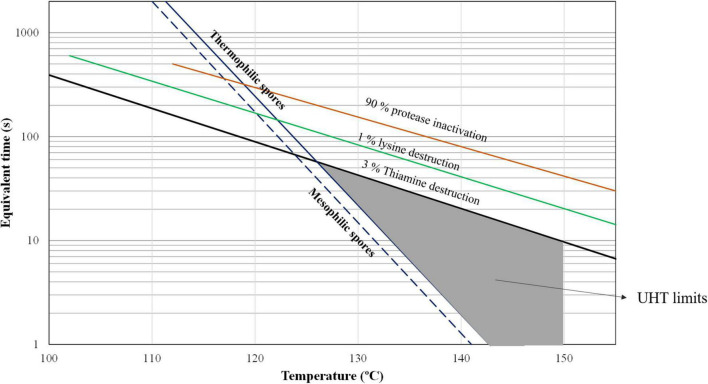
Effect of temperature and equivalent time on the lethality of common spores in raw milk, indicating the UHT conditions to produce safe long-life milk [adapted from Bylund et al. (2)].

Because of the high temperatures applied to milk in UHT, the heat-resistant spores are destroyed as shown in Figure 2. Since it is necessary to prevent recontamination, the product is packaged under aseptic conditions after treatment. As a consequence, the shelf-life of UHT milk is long at ambient temperature.

Concerning nutritional aspects, according to Bylund (2), there are no changes in the nutritional values of fat, lactose, and minerals, but there are marginal changes in the nutritional value of proteins and vitamins. In Figure 2, it can be observed the combinations of the temperature-equivalent time highlighted in the UHT region, that most of thiamine and lysine are preserved.

Both HTST and UHT treatments are widely applied to other fluid foods by the industry. For example, the liquid egg is a low-acid food for which the pasteurization parameters are regulated by legislation concerning *Salmonella* spp. On the other hand, for high-acid or acidified foods, the combination of time and temperature is dependent on the pH to provide a shelf-stable product. For acidified or natural products with pH < 4.0, it is recommended 1 min at 87.8°C, whereas for a product with pH = 4.0, 30 s at 96.1°C; and for pH = 4.1, 30 s at 100°C. Therefore, it can be concluded that as the food is less acid, a more intense heat treatment is needed. However, if other substances are present, like sugar or starch, the conditions can change (7). In summary, for a particular food, it is necessary to design adequate thermal processing conditions, to know the potential microorganisms (pathogens and spoilage), enzymes, and composition, to ensure a safe product under the health public interest, with high quality.

For fruit juices, flash pasteurization or HTST is quite used by the industry. The juice is submitted to very fast heating until the desired temperature is achieved, followed by a necessary holding time, cooling, and aseptic filling. Again, the parameters of the pasteurization are established according to the pH: for peach juice (pH < 4.5) the holding time is 30 s at 110°C; for orange juice is 1 min at 90 °C or 30 s at 95°C; for grapefruit is 16 s at 74°C or 1 s at 85°C. It is important to say that it is mandatory, for juices, by the FDA regulations, to design the process to achieve a minimum of 5 log reduction of pathogenic microorganisms (7).

The consumption of processed foods has been systematically criticized based on classifications such as the NOVA classification (8) because they mix food composition and nutritious value with the unit operations required for processing, which lead to the idea that unprocessed food or homemade prepared food are better options. Food processing is not only about shelf life and palatability since it provides consumer safety by inactivating harmful pathogens while preserving quality (5). For instance, boiling milk at home is a harsher condition than UHT industrial processing on the nutrients of milk because of the different levels of energy.

Another important aspect is that there are several ongoing types of research concerning emerging and sustainable technologies to address consumer demands. Novel and improved thermal processes are presented as the use of superheated steam, ohmic heating, microwave-assisted processing, showing improved retention of nutrients. Moreover, non-thermal physical processes are also under development, such as pulsed electric field (PEF).

Microwave-assisted processing has been widely explored, among others, for pasteurization, sterilization, drying, or thawing of food products. Many industries use microwaves in combination with conventional thermal methods. In the food industry, the support of microwaves reduces the processing time and energy consumption, while retaining the sensory quality and causing minimal changes in the nutritional value of food compared to using only conventional methods (9).

So far, we mostly discussed the thermal processing of foods under continuous conditions. No less important is the batch processing of food packed in cans, retortable pouches, rigid plastic containers, or glass containers. Since Appert’s experiments, studies have been conducted to improve predictive methods to determine the temperature history inside the package at the point of slowest heating (cold spot) during heating and subsequent cooling.

As with the continuous process, the parameters for the batch thermal processes are established according to the pH and nature of the food. Furthermore, as the heating inside packaged food is not uniform, and because it allows for an anaerobic environment, the concern with the development of *Clostridium botulinum* has resulted in very strict regulations, to prevent poisoning. *C. botulinum* is an anaerobic mesophilic microorganism, whose spores can germinate, grow and produce the lethal botulinum toxin. For canned foods, mainly those low-acid (pH > 4.6), the thermal process should be designed to achieve the commercial sterility, that is the regulatory agencies recommend a 12 decimal reduction of the target microorganism, resulting in a probability of survival of 10^–12^.

Commercial sterility is a more severe thermal process to eliminate all vegetative species and spores that can develop at storage conditions after the process. Besides food safety, this process provides a stable product at room temperature, if the package can guarantee no post-contamination.

Fortunately, spoilage microorganisms often are more heat resistant than *C. botulinum*, and consequently, they are considered the process target. To be reasonably sure of the safety of a process, a specific food may be inoculated with an organism with known heat resistance (and more heat resistant than *C. botulinum*), processed under controlled conditions, and the extend of spoilage compare to the probability of spoilage design into a process. This is accomplished with the lethality calculated from the temperature history obtained experimentally, through a measured temperature at the cold spot in real-time.

To illustrate, the heat resistance of *C. botulinum* in low-acid foods can vary from 0.22 min (in green beans) to 2.33 min (in spinach) at 121.1°C, that is, depending on the media (7). It is known that the spores are unable to germinate at pH below 4.6. For this reason, for some kinds of food, which are sensitive to heat in severe conditions, acidification is used by the industry to provide a safe product.

D’Incecco et al. (10) in their review present an overview of novel technologies for milk processing, some under research or commercially available. The objectives of these alternative technologies are to achieve microorganisms and enzymes inactivation efficiently with minimum damage of milk components. Among the non-conventional heat treatments, they highlighted ohmic heating, microwave heating, and radio-frequency heating. Among non-thermal technologies, to produce higher quality food, they cited microfiltration, high-pressure processing, pulse-electric field, and ultrasound.

## Conclusion

In this opinion paper, we seek to show the reader the importance of unit operations to produce safe and high-quality food. Although many unit operations applied in the food industry are similar to culinary practices, effective control and scientific knowledge about the fundamentals involved are incomparable in terms of the high quality achieved. With the progress of research in several centers around the world, the scientific community together with the development centers of the industry have been developing and improving new technological alternatives to meet the current consumer demand for healthier foods, without giving up on safety. It was shown in this text how the evolution of knowledge contributed to what we know today as thermal food processing, which allows the consumption of pasteurized or sterilized foods without causing disease.

## Author Contributions

CT and JG: conceptualization, investigation, resources, writing – original draft, and writing – review and editing. Both authors contributed to the article and approved the submitted version.

## Conflict of Interest

The authors declare that the research was conducted in the absence of any commercial or financial relationships that could be construed as a potential conflict of interest.

## Publisher’s Note

All claims expressed in this article are solely those of the authors and do not necessarily represent those of their affiliated organizations, or those of the publisher, the editors and the reviewers. Any product that may be evaluated in this article, or claim that may be made by its manufacturer, is not guaranteed or endorsed by the publisher.
